# Finding the Balance: Fertility Control for the Management of Fragmented Populations of a Threatened Rock-Wallaby Species

**DOI:** 10.3390/ani5040414

**Published:** 2015-12-16

**Authors:** Nicole Willers, Graeme B. Martin, Phill Matson, Peter R. Mawson, Keith Morris, Roberta Bencini

**Affiliations:** 1 School of Animal Biology, The University of Western Australia, M092, 35 Stirling Highway, Crawley, WA 6009, Australia; E-Mails: nicole.willers@dpaw.wa.gov.au (N.W.); graeme.martin@uwa.edu.au (G.B.M.); 2 Department of Parks and Wildlife, Locked Bag 104, Bentley Delivery Centre, WA 6983, Australia; E-Mail: keith.morris@dpaw.wa.gov.au; 3 Perth Zoo, South Perth, WA 6151, Australia; E-Mails: phill.matson@fertilitynorth.com.au (P.M.); peter.mawson@perthzoo.wa.gov.au (P.R.M.)

**Keywords:** Rock-wallaby, *Petrogale lateralis*, deslorelin, fertility control, wildlife management, overabundance, free-living, hormonal implants

## Abstract

**Simple Summary:**

Black-flanked rock-wallabies (*Petrogale lateralis lateralis*) can reach high numbers in fragmented populations in the West Australian wheat-belt, where they can damage crops and cause habitat degradation. As they are threatened, we wanted a non-permanent control method that did not adversely affect the body condition of treated females compared to untreated females, using body condition as an indicator of general health and fitness. We gave adult female rock-wallabies deslorelin contraceptive implants to suppress their fertility and monitored the impact for three years. Treated females did not conceive new young for over two years. We did not detect any negative effects on body condition, suggesting that deslorelin may be an effective tool for managing overabundant populations of marsupials.

**Abstract:**

Populations of Australian marsupials can become overabundant, resulting in detrimental impacts on the environment. For example, the threatened black-flanked rock-wallaby (*Petrogale lateralis lateralis*) has previously been perceived as overabundant and thus ‘unwanted’ when they graze crops and cause habitat degradation. Hormonally-induced fertility control has been increasingly used to manage population size in other marsupials where alternative management options are not viable. We tested whether deslorelin, a superagonist of gonadotropin-releasing hormone (GnRH), would suppress reproduction in free-living adult female rock-wallabies without adversely impacting body condition. We trapped, synchronised reproduction and allocated female rock-wallabies to a placebo implant (control, n = 22), one (n = 22) or two (n = 20) subcutaneous implants of deslorelin. Females were then recaptured over the following 36 months to monitor reproduction, including Luteinising Hormone levels, and body condition. Following treatment, diapaused blastocysts reactivated in five females and the resulting young were carried through to weaning. No wallabies treated with deslorelin, conceivede a new young for at least 27 months. We did not observe adverse effects on body condition on treated females. We conclude that deslorelin implants are effective for the medium-term suppression of reproduction in female black-flanked rock-wallabies and for managing overabundant populations of some marsupials.

## 1. Introduction

The macropod marsupials, a group that includes the wallabies and kangaroos, are the largest and most visible component of the Australian mammal fauna [1]. Macropods often become overabundant when human interference leads to artificial isolation, provision of supplementary resources, control of predators and/or habitat protection [2,3,4]. Many kangaroo and wallaby populations are managed because of perceived competition with livestock, crop grazing, and their contribution to the degradation of native ecosystems [3]. For example, in environments modified for agriculture, the size of kangaroo populations can increase by up to 44% each year [2]. This level of expansion can have considerable economic and environmental effects [5,6,7]. Furthermore, animal welfare concerns arise in low rainfall years when large numbers of animals searching for food increase the likelihood of collisions with vehicles [8].

High densities of macropods have historically been managed by culling (*i.e*., shooting, poisoning), but this approach attracts intense public attention and increasing opposition, and is not appropriate for species of conservation concern [9]. Hormonal fertility control is one of the non-lethal alternatives being investigated [10,11,12]. A non-permanent form of control may be desirable for small, accessible populations that are highly valued because control can be ceased if required, and genetic diversity is more readily maintained [10]. One product that shows promise for marsupials is deslorelin, a Gonadotropin Releasing Hormone (GnRH) superagonist, that temporarily blocks reproduction in a variety of species such as brushtail possums (*Trichosurus vulpecula*), eastern and western grey kangaroos (*Macropus giganteus, M. fuliginosus*) and tammar wallabies (*M. eugenii*), with no evidence of negative side-effects on body condition [11,13,14,15,16]. However, some major issues need to be resolved before deslorelin can be routinely adopted:

(a) wide between-animal variation in the duration of infertility was detected in tammar wallabies and grey kangaroos [11,13,15] that may be related to individual differences in sensitivity to GnRH agonist-induced desensitization [17];

(b) small sample sizes, usually less than 10 per treatment group, limit the generality of the studies [11,13,15,18];

(c) the validity of application to management of free-living populations is not clear because most published studies used captive or semi-captive marsupials where the population structure, animal nutrition, and seasonal and social influences were managed to some extent [11,13,14,15,16];

(d) the risk that the treatment will be ineffective because of subtle differences between species and sexes in the pituitary response to GnRH superagonists [19], although such issues seem to be limited to males [16,20];

(e) there are also three risk factors that may raise animal welfare concerns: (i) potential unwanted side-effects flowing from decreased reproductive function and sex steroid production, e.g., disruptions of an individual’s position in a social hierarchy that might cause stress and loss of body condition [10,21,22]; (ii) disruption of blastocyst reactivation, pregnancy, birth or the initiation of lactation [11]; and (iii) the failure of deslorelin-treated females to re-breed within the expected timeframe [11,15,16,18] indicating the potential for deslorelin to cause permanent infertility in some individuals [14].

A good candidate species for addressing these issues is the black-flanked rock-wallaby (*P.lateralis lateralis*), which occurs in widely disjunct populations in Western Australia, but has declined in distribution and abundance and is listed as ‘near threatened’ under IUCN criteria at the species level, but Vulnerable at the sub-species level on Western Australian and national threatened species listings [23,24,25,26,27]. Black-flanked rock wallabies are thought to have an average life span of 5–10 years, but some individuals are known to have lived for up to 12 years.

Many populations were threatened with extinction due to predation by the European red fox (*Vulpes vulpes*) but recovered with the implementation of fox control in the 1980s [28]. Two populations experienced such a large expansion following predator control that they became overabundant and caused economic damage to neighbouring properties, and contributed to the degradation of habitat in the reserves they inhabit [29,30]. That is, effecting a reduction in vegetation cover, plant biomass and plant health from overgrazing and impairing the ability of plants to set seed and reproduce. Interventionist management has been necessary in these cases. To reduce population sizes, a large number of animals were removed for translocations, but when additional suitable translocation sites could not be identified, non-permanent fertility control had to be investigated for managing the rock-wallaby population [31].

We tested whether deslorelin can prevent reproduction in females in a free-living population of black-flanked rock-wallabies, and used larger treatment groups than previous studies to provide a robust data set so that we could assess the variability in the mean duration of contraception. We expected that a single implant (4.7 mg) would suppress reproduction for at least 515 days following the results of a study on a similar sized macropod, the tammar wallaby [13]. We also tested whether the use of two deslorelin implants, simultaneously, would reduce variation in the period of reproductive suppression. Double doses (9.4 mg) of deslorelin have been used to achieve longer periods of fertility control, which may be advantageous in free-living populations where a cost-benefit balance is important [32]. Finally, we tested whether deslorelin treatment would affect body condition, as an indicator of general health and fitness, or cause any other undesirable effects that might affect reproduction.

## 2. Materials and Methods

### 2.1. Study Site

**Figure 1:**
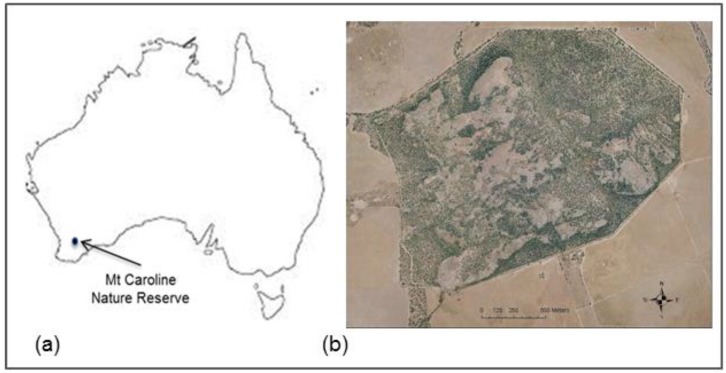
**(a**) Map of Australia showing the location of Mt Caroline Nature Reserve; (**b**) Aerial photograph of Mt Caroline Nature Reserve, showing reserve boundaries surrounded by farmland. Bare areas within the reserve are smooth-faced granite rock.

The *P. l. lateralis* population used in this study was located within Mount Caroline Nature Reserve (−31°47'24" S. 117°38'6" E.), a 350 ha granite outcrop, in the central wheat belt of Western Australia (Figure 1) as detailed elsewhere [31,33]. During the study, the condition of the vegetation in the reserve was poor, with very little recruitment and considerable senescence and grazing damage to shrubs. Rock-wallabies shelter in caves and complex rock structures during daylight hours, emerging at sunset to feed in and around the rocks or venture out into grasslands.

### 2.2. Trapping Protocol, Animal Handling and Data Collection

All procedures complied with the National Health and Medical Research Council’s “Australian code of practice for the care and use of animals for scientific purposes” [34], were approved by the Animal Ethics Committee of The University of Western Australia (07/100/577) and were conducted under the provision of Department of Environment and Conservation scientific purposes licences (SF006153, SF005628, SF006668).

We captured *P. l. lateralis* using soft-walled ‘Thomas’ traps over nine trapping sessions from April 2007 to April 2010 as reported elsewhere [31]. Intervals between trapping sessions ranged from two to six months. Captured females were classified based on their reproductive status as: immature (sub-adult with no pouch young, small pouch, inverted teats); mature with no pouch young (adult, developed pouch, everted teats); lactating (adult with no pouch young present but with an elongated teat); or mature with pouch young (when a pouch young was present). The weight of pouch young was subtracted from that of their mothers to determine the weight of adult females [31].

In April 2007, we captured free-living adult (>2500 g) female rock-wallabies and randomly allocated them to one of three treatments: a placebo implant (n = 22); a single (4.7 mg) deslorelin implant (n = 22); or two deslorelin implants loaded into the implanting device simultaneously (9.4 mg, n = 20). All captured females received unique identification tags so that they could not receive two implants and could then be monitored for the duration of the study. Neonatal pouch young less than 60 days old were humanely euthanased using pentobarbital sodium injections (Lethabarb, Virbac Australia) to synchronise the reproductive cycles of the adult females. Females carrying pouch young 60 days and older, determined as outlined in Section 2.6 below, were excluded from the experiment. The three groups contained about equal numbers of females with neonatal young that needed to be euthanased (9.4 mg = 14, 4.7 mg = 12, placebo = 10). As the rock-wallabies were free-living, there was no certainty of recapture.

During subsequent monitoring, pouches were checked for the presence of pouch young. It is possible that breeding events could have been missed during the gaps between trapping sessions, but only if pouch young were carried for a short time and then lost. Trapping session gaps were not long enough to miss young that were carried through full pouch life. The age of pouch young was estimated using head and pes (the terminal segment of the hind-limb) length and developmental features (eyes, ears and body), by comparison with growth rates of pouch young of *P. l.* MacDonnell Ranges race [35]. Although the growth rate was based on a different race, it was the best estimate available, and is likely to be similar because Jones, Taggart and Temple-Smith [35] found no difference in pouch young development between *P. l.* MacDonnell Ranges race and *P. l. pearsoni* indicating that pouch young develop similarly in the different races of the species. The estimated age of the pouch young was then subtracted from the date the animal was captured to provide a date of birth of the pouch young. Length of gestation was assumed to be 30 days [27]. We could therefore estimate the date on which the deslorelin implant ceased being effective.

### 2.3. Deslorelin Implantation

The 2.3 mm × 12.5 mm cylindrical implants (Suprelorin; Peptech Animal Health Pty Ltd, Macquarie Park, Australia) each contained 4.7 mg deslorelin (D-Trp^6^-Pro^9^-des-gly^10^-GnRH ethylamide) and were pre-packaged in a purpose-developed disposable implanter that was sterilized by e-beam irradiation and provided by the manufacturer. Placebo implants were made of the same extruded lipid base but contained no deslorelin. Implants were placed subcutaneously between the shoulder blades. The injection site was sealed with a veterinary skin adhesive (Vetbond; 3M Animal Care Products, St Paul, MN, USA).

### 2.4. Blood Sampling and GnRH Challenges

To determine the efficacy of pituitary desensitization by deslorelin we administered exogenous GnRH to all implanted rock-wallabies captured during all trapping sessions except the first and last [36]. Deslorelin suppresses pituitary function and we expected that treated females would show no hormonal response but placebo females would show a rise in Luteinising Hormone (LH) after the GnRH challenge. Females that were recaptured with large pouch young during subsequent trapping sessions were excluded because the stress could have caused the mother to abandon the young. Synthetic GnRH (Fertagyl, Intervet Pty Ltd., Victoria, Australia; 2.5 µg/kg) was delivered intramuscularly in the hind leg. Jugular blood (0.5–2 mL) was sampled 5 minutes before GnRH administration and 25 minutes later [35]. Blood was immediately transferred into collection tubes (Vacuette, EDTA, Greiner Bio-one, Austria) and centrifuged at 3000 rpm. The plasma was separated and stored at −20 °C until assayed to determine the concentration of Luteinising Hormone (LH).

### 2.5. LH Assays

The concentration of LH was measured in each plasma sample with an enzyme-immunoassay using a mono-clonal antibody against anti-bovine LH that has been validated for *P. l. lateralis* [37]. The limit of detection was 0.86 ng/mL, and the inter-assay coefficients of variation for three quality control pools containing 1.24 ± 0.08, 2.51 ± 0.15 and 5.30 ± 0.43 ng/mL (means ± S.D.) were 6.42, 5.80 and 8.14%. The intra-assay coefficient of variation was 7.9% at 0.94 ng/mL, 10.2% at 2.5 ng/mL and 6.1% at 9.4 ng/mL. Results are expressed in terms of the bovine LH standard.

### 2.6. Statistical Analyses

The duration of infertility was calculated as the number of days between when the treatment or control was administered, and the estimated date each female gave birth to their next detected young. We assessed the effect of treatment on the duration of infertility using a Kaplan-Meier estimator with a log-rank (Mantel-Cox) [38,39]. The Kaplan-Meier analysis allows the inclusion of ‘censored’ data, an appropriate approach for rock-wallabies that died, were not recaptured or did not breed before the end of the study.

Using pes length, we calculated a measure of adult body condition using the deviation of its actual body mass from the average, given its size, to assess the general health and fitness of individuals over time [31]. Body condition is significantly associated with total gastrointestinal parasite burden, and many serum biochemistry and hematology analytes in tammar wallabies (*M. eugenii*), demonstrating body condition as a reliable indicator of general health [40]. We used a linear mixed model to account for variation within and between rock-wallabies, where rock-wallaby identity (ID) was included as a random effect. In the model, the response of body mass to the fixed effects of pes length (covariate) and treatment was investigated. The residuals from the model provided a body condition index that was specific for each individual and relative to its own ‘usual’ condition [41]. We tested for a relationship between the condition index, treatment and trapping session using a linear mixed model. Trapping session and treatment were included in the model as fixed effects, and ID as a random effect to account for any correlation between the repeated measurements of the same individual over time [31].

To analyse the results of the GnRH challenges, we first calculated the difference in LH concentrations between samples collected prior to the GnRH challenges and those 25 min later. We then used a linear mixed model to test for a relationship between the difference in LH concentrations and the fixed effects of treatment and trapping session, with ID as a random effect.

All results are presented as means plus or minus their standard errors.

## 3. Results 

### 3.1. Births

**Figure 2:**
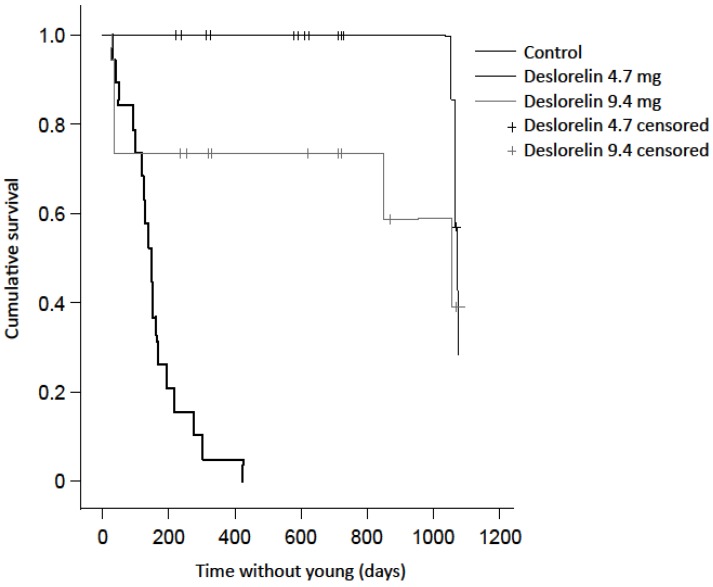
Kaplan Meier survival plot of the contraceptive effect illustrated by the time without young between treatment groups.

Deslorelin implantation prevented reproduction in treated females. Time without young was significantly less in control females (160 ± 22 days, n = 19) than those treated with a single (1073 ± 5 days, n = 20, *X^2^* = 45.4, *p* < 0.001) or double (770 ± 106 days, n = 19, *X^2^* = 15.3, *p* < 0.001) deslorelin implant. The first pouch young detected following treatment with deslorelin was conceived 818 days (27 months) after treatment. The double implant group included five animals that reactivated blastocysts to give birth to a new young within 36 days of the contraceptive treatment, but were not known to re-breed again within the timeframe of the study. The inclusion of these ‘outliers’ did not affect the significant result of the log rank test. When these outliers were removed, the mean duration without young increased to 1033 ± 42 days. There was no difference in time without pouch young between the single and double deslorelin implant groups, including the outliers (*X^2^* = 3.1, *p* = 0.080; Figure 2).

As the rock-wallabies were free-living during the study, not every animal was caught in every monitoring session. Of the 22 females that received a placebo implant, all were recorded with a new young except for three that were not recaptured after receiving the implant. Of the 22 females that received a single deslorelin implant, only four were known to have re-bred before the end of the study, and two were not recaptured following implantation. Of the 20 that received two implants, only seven were known to have re-bred before the end of the study, and only one was not recaptured following implantation (Table 1).

**Table 1:** The outcomes for female rock-wallabies treated with a placebo implant (n = 22), or one (n = 22) or two deslorelin implants (n = 20).

Treatment	Placebo	One Implant	Two Implants
Recaptured with new young during study	19 (86.4%)	4 (18.2%)	7 (35 %) *
Not recaptured following implantation	3 (13.6%)	2 (9.1%)	1 (5 %)
Recorded in final year without young	n/a	8 (36.4%)	5 (25 %)
Not recaptured in final year	n/a	8 (36.4%)	7 (35 %)

* Includes five females that re-bred within an estimated 36 days of implantation.

### 3.2. Luteinising Hormone Concentrations

**Figure 3:**
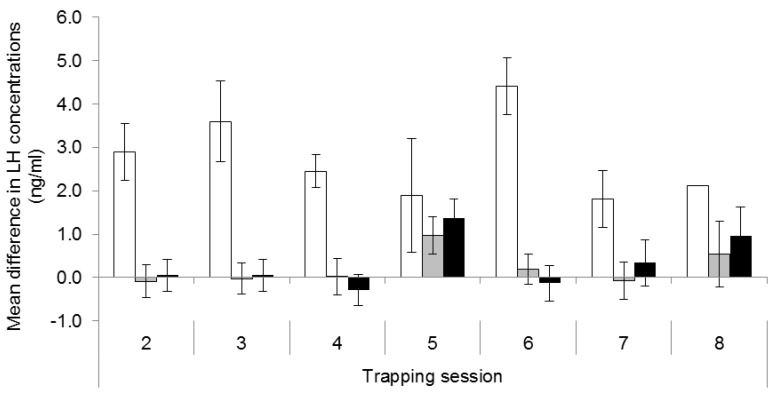
Mean difference in plasma concentrations of Luteinising Hormone (LH) (ng/mL ±SE) over trapping sessions between treatment groups (white = placebo; grey = single deslorelin implant; black = double deslorelin implant). Sample sizes by treatment group in order of time are: placebo (8, 2, 12, 2, 4, 4 and 1) single deslorelin implant (13, 14, 10, 9, 14, 10 and 3) and double deslorelin implant (13, 13, 14, 10, 10, 6 and 4).

The increase in LH concentrations between blood samples taken before and after GnRH challenges differed among treatments (F_2,168_ = 42.31, *p* < 0.001) but not trapping sessions (F_6,168_ = 1.23, *p* = 0.294). The rise in LH was greater in control females (2.73 ± 0.34 ng/mL) than in the females with one (0.22 ± 0.18 ng/mL, *p* < 0.001) or two implants (0.34 ± 0.17 ng/mL, *p* < 0.001) over time, and there was no difference between single and double implant groups (*p* = 0.624; Figure 3). However, the deslorelin treatment did not completely block the response to exogenous native GnRH. In the double implant group, there was a small response (*p* = 0.01) in August 2008 (Figure 3) when three females displayed a marked increase in LH concentrations. Of those females, two had no pouch young during subsequent recaptures and did not show increased LH concentrations in any further GnRH challenges, and one female was not recaptured following August 2008.

The first deslorelin-treated female to be recaptured with a pouch young, at Trapping Session 8, responded to the GnRH challenge at Trapping Session 7. Of the other five deslorelin-treated females that re-bred before the end of the study, only one responded to a GnRH challenge during Trapping Session 8 and was detected with a pouch young at Trapping Session 9. Three of these five females were not recaptured during Trapping Session 8 and therefore were last sampled at Session 7 or earlier.

### 3.3. Body Condition

**Figure 4:**
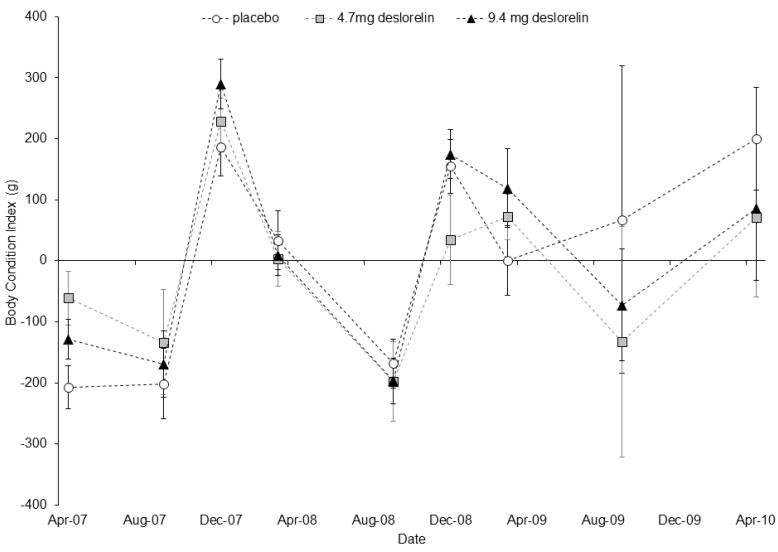
Variation in the average body condition index (±SE) over time between treatment groups (white circle = placebo; grey square = single deslorelin implant; black triangle = double deslorelin implant) of *P. l. lateralis* females. Sample sizes by treatment group in order of time are: placebo (19, 7, 15, 14, 6, 11, 12, 3 and 6) single deslorelin implant (19, 8, 13, 13, 9, 13, 10, 3 and 3) and double deslorelin implant (20, 16, 15, 14, 10, 10, 9, 4 and 3). Note: the December 2009 data point is missing.

Body mass was significantly related to pes length (F_1,231_ = 249.66, *p* < 0.001) but not to treatment (F_2,42_ = 1.07, *p* = 0.352). The pes x treatment interaction was not significant and was therefore excluded from the model. Body condition index varied significantly with trapping session (F_8,274_ = 23.19, *p* < 0.001) but not with treatment (F_2,274_ = 0.81, *p* = 0.447). The trapping session x treatment interaction was not significant and was therefore excluded from the model. The condition of control and treated females fluctuated annually and condition was generally highest in early summer (December 2007 and 2008), and lowest in early spring (September 2007, 2008 and 2009; Figure 4). Overall, body condition was highest in December 2007 and lowest in September 2008.

## 4. Discussion 

Our study demonstrates the efficacy of deslorelin implants for medium-term suppression of reproduction in free-living female rock-wallabies. There were no adverse effects on body condition, suggesting the treatment did not affect general health and fitness of the animals, yet conception was delayed for at least 818 days (27 months). Importantly, using two implants instead of one did not improve the outcome, and did not reduce the variability in the duration of infertility.

### 4.1. Efficacy of Deslorelin Implants

The presence/absence of pouch young and the response to GnRH challenges, confirmed the effectiveness of deslorelin as a treatment for blocking gonadotropin secretion and therefore ovulation in *P. l. lateralis.* The time without young, a measure of the duration of contraception, was extended from 160 days (placebo) to over 1000 days. A single 4.7 mg implant would be the best approach to suppress reproduction and control fertility in overabundant populations of rock-wallabies.

Use of two deslorelin implants simultaneously offered no advantage over a single implant as in both cases, the LH response to GnRH and fertility were blocked. The double dose also did not provide any advantage with respect to duration of infertility. This is consistent with the nature of such implants because they lose efficacy when sufficient material has been dissolved and the dose of active ingredient falls below a threshold where pituitary secretion of gonadotropins is impeded. Two individual implants would dissolve at the same rate as one because the rate of dissolution is based simply on surface area. To achieve longer periods of suppression would require either a larger implant that minimises the surface area/volume ratio, or the administration of a second implant about 800 days after the first. These options need to be tested in future research.

The occurrence of embryonic diapause in rock-wallabies is an important consideration in the timing of interventions for fertility control. If deslorelin does not affect the reactivation of blastocysts in rock-wallabies, the effective period of reproductive control is reduced. Instead of achieving at least 27 months of suppression, deslorelin may only provide 20 months control, assuming gestation is approximately one month and pouch life 6–7 months [27]. This may be reduced even further if a pouch young is present at the time when deslorelin is administered, and a successful *post-partum* mating has already occurred, resulting in a diapaused embryo. If the pouch young is only one month old, it may have 6 months of pouch life remaining, and the following embryo will take approximately 8 months, from gestation to weaning. Therefore, the 28 months of potential suppression may be reduced to 14 months. However, if the existing pouch young was removed at the time deslorelin was administered, provided it was of an acceptable size to do so, this would minimize the reduction in efficacy. *P. l. lateralis* reproduce throughout the year but have two peaks of reproduction, one in autumn and one in late winter/spring [31]. Deslorelin implants would be most effectively delivered at these times if pouch young were removed, as the young would be sufficiently small (e.g., neonatal) to allow for humane disposal. If removing pouch young was undesirable, deslorelin implants would best be administered shortly before these times to prevent conception in the majority of females. If deslorelin was delivered while a pouch young was present, further research would be required to determine whether the implant would affect the growth or development of pouch young, as the sexual maturation of female tammar wallaby pouch young whose mothers were treated with deslorelin was significantly delayed [17].

Five females from the double implant group re-bred within an estimated 36 days of receiving deslorelin, almost certainly because diapaused blastocysts were present and reactivated following the removal of pouch young when the treatment was administered [42,43,44,45,46]. Herbert, Trigg and Cooper [11] suggested that successful blastocyst reactivation and birth after deslorelin treatment was unlikely because some aspect of blastocyst reactivation, pregnancy, birth or the initiation of lactation was presumably affected by deslorelin in tammar wallabies. It is unclear what proportion of the double implant group would have been carrying diapaused embryos and therefore, whether those five represent a high or low rate of successful reactivation. However, they do suggest that deslorelin treatment does not affect blastocyst reactivation, pregnancy, birth or the onset of lactation in rock-wallabies.

There is a wide variation in the duration of contraception using deslorelin implants among individual *P. l. lateralis,* as has been found in other species [47,48,49]. Thirty-one percent of all treated females still had no young in the final year of the study, suggesting that the efficacy of the deslorelin treatment may have been considerably longer than the duration of this study, or that deslorelin caused a semi-permanent disruption of the reproductive cycle in many of the females that failed to re-breed. The release of deslorelin is likely to become highly variable as the implants disintegrate over time, causing the wide variation observed in this and other studies.

### 4.2. Potential Risks of Deslorelin Implants

Deslorelin implants did not adversely affect the body condition of treated females which instead, varied seasonally. Across the total population, this seasonal fluctuation in body condition was closely related to rainfall in the period ~3–6 months before trapping sessions [31]. For example, winter rainfall was the best predictor of body condition during early summer as good winter rains result in greater food biomass in the following spring, allowing the rock-wallabies to gain condition [31,50]. Individual condition has been reported to improve as a result of fertility control [51], but we did not detect this, possibly because resource availability across the population was poor and this may have limited the ability of treated females to improve their condition.

Although deslorelin did not adversely affect body condition, it is possible that by altering reproductive function in some females, deslorelin disrupted the social hierarchy. Female rock-wallabies in this population displayed strong philopatry and there are clusters of related females that could include social hierarchies [52]. However, the very high rates of reproduction detected from the beginning of this study, before deslorelin was applied, suggest that social dominance in breeding is unlikely. Despite this, future research should assess the impact of infertility on behaviour at an individual and population level, to determine the impact on any social structuring.

Although some of the deslorelin-treated rock-wallabies re-bred within the timeframe of this study, about two-thirds (28/42) did not. However, only about one-third (13/42) were recaptured in the final year. We do not know for how long these individuals remained infertile and whether any were rendered permanently infertile. In managing a threatened species such as *P. l. lateralis* it is important that any fertility control does not result in permanent infertility. Additional research is therefore necessary to determine the total duration of deslorelin-induced contraception in all females, and to ensure that deslorelin does not cause permanent infertility in any individuals.

## 5. Conclusions 

Our results show that deslorelin implants can be used to temporarily suppress reproduction in free-living female black-flanked rock-wallabies. For some females at least, sterility was not permanent and there was no impact on body condition. The successful birth and persistence of young in the pouch of treated females suggest that deslorelin treatment does not affect blastocyst reactivation, pregnancy, birth or the onset of lactation.

Two major issues still need some resolution: (i) optimal timing of the initial deslorelin implantation, because embryonic diapause will reduce the duration of infertility to some extent; (ii) and the timing of re-treatment, if required, because a single (4.7 mg) deslorelin implant will suppress reproduction for only 27 months. Deslorelin is, nevertheless, an effective tool for managing populations of black-flanked rock-wallabies and, possibly, other rock-wallaby and marsupial species where overabundance has become a problem.
